# An Appreciation

**Published:** 1911-10-21

**Authors:** Leonard D. Rea

**Affiliations:** King Edward VII.'s Hospital, Cardiff


*To the Editor of* The Hospital.

### AN APPRECIATION.

*To the Editor of* The Hospital.

Sir,—May I take this opportunity to congratulate you on the improved Hospital? I believe it will now prove of even greater service to our institutions generally, as it should reach a larger circle of readers interested in hospital and institutional life.—Faithfully yours,

Leonard D. Rea.

King Edward VII.'s Hospital,
Cardiff, October 16.

